# Long follow-up treating CHILD nevi with topical cholesterol and statins^☆^

**DOI:** 10.1016/j.abd.2025.501280

**Published:** 2026-01-29

**Authors:** Milene Tiburcio Narenti Ferradoza, Ana Clara Maia Palhano, Julia Maria de Oliveira Neumayer, Luciana Paula Samorano, Maria Cecilia Rivitti-Machado, Zilda Najjar Prado de Oliveira

**Affiliations:** Department of Dermatology, Hospital das Clínicas, Faculdade de Medicina, Universidade de São Paulo, São Paulo, SP, Brazil

Dear Editor,

CHILD nevi comprise the cutaneous manifestations typical of CHILD syndrome, an acronym for Congenital Hemidysplasia with Ichthyosiform Erythroderma and Limb Defect, a rare, X-linked dominant genetic disease, lethal to males, resulting from mutations in the NSDHL gene, which belongs to the cholesterol biosynthesis pathway.^1^

Altered cholesterol biosynthesis affects myelin formation and organogenesis, resulting in neurological, skeletal, and visceral malformations.^2^, ^3^ CHILD nevi arise as a physiological compensation for the lipid-poor and inflamed skin barrier due to the accumulation of its precursors, resulting in erythematous-squamous plaques that follow Blaschko's lines, with a predilection for fold areas, associated with pruritus and recurrent skin infections, which affects quality of life and represents a potential life-threatening risk.^1^, ^4^

Laboratory tests are usually normal, and histopathological examination of CHILD nevi reveals nonspecific psoriasiform changes, which are useful for ruling out differential diagnoses. Clinical evaluation and, when available, genetic testing confirm the diagnosis.^1^

The first treatments used for CHILD nevi were topical keratolytic agents, emollients, corticosteroids, and calcineurin inhibitors, oral retinoids or methotrexate, showing unsatisfactory results, in addition to representing risks inherent to prolonged use and in potentially fertile patients.^5^ Therapeutic proposals based on the disease pathogenesis provide more satisfactory results and include the topical association of cholesterol with statins, aiming to replace the deficient lipid while inhibiting an initial phase of cholesterol biosynthesis, preventing the accumulation of potentially toxic sterols and other mediators.^6^, ^7^, ^8^, ^9^

The present case describes the long-term follow-up (1 to 23 years) of five female patients with CHILD Syndrome in the Pediatric Dermatology outpatient clinic of Hospital das Clínicas, Faculty of Medicine, Universidade de São Paulo, Brazil.

All five cases had CHILD nevi, with pruritus and recurrent infections, which improved when the lesions became thinner and less inflamed. Ipsilateral limbs' hypoplasia with CHILD nevi was the rule; in Case 5, there was aplasia of the right upper limb. Three of the five patients have the left side affected (1, 3, and 4), with cases 1 and 3 having more associated malformations (Table 1). Studies suggest that the right side is more affected (7/3 ratio), but patients with left-sided involvement tend to have a worse prognosis, as visceral abnormalities are more common.^1^, ^2^, ^4^

**Table 1:** Comparative analysis of the clinical characteristics and therapeutic response of 5 cases of CHILD Syndrome.

	Case 1	Case 2	Case 3	Case 4	Case 5
Patient's age	24 years	12 years	8 years	2 years	1 year and 9 months
Time of follow-up	23 years	12 years	8 years	2 years	9 months
Affected side of the body	Left	Right	Left	Left	Right
Predilection for skin lesions in fold areas	Yes	Yes	Yes	Yes	Yes
Discomfort with pruritus and infections in CHILD nevi	Yes	Yes	Yes	Yes	Yes
Limb hypoplasia or agenesis	UL + LL	UL + LL	LL	UL + LL	UL + LL
Other malformations	Scoliosis	Hearing loss	Ovoid vertebral bodies	No	Scoliosis
	Hearing loss		Craniosynostosis Left pyelic ectasia		
			Patent foramen ovale		
Treatment with 2% Lovastatin + 2% topical Cholesterol	6 years	9 years	8 years	No	No
Treatment with 2% Simvastatin + 2% topical Cholesterol	4 years	8 months	No	2 years	9 meses
Treatment with 2% Atorvastatin + 2% topical Cholesterol	No	1 year and 6 months	No	No	No
Skin clinical stability	No	Yes	Yes	Yes	Yes
Improvement in quality of life and pruritus with treatment.	Yes	Yes	Yes	Yes	Yes

UL, Upper Limb; LL, Lower Limb.

The two longest-standing patients in the follow-up (Cases 1 and 2) used topical keratolytic agents (10% urea, occlusive salicylic acid), emollients, and calcipotriol at the beginning of treatment, with an unsatisfactory therapeutic response. Cutaneous therapy with lovastatin and cholesterol showed a satisfactory response in four of the five patients.

The average time required to obtain a satisfactory response with therapy based on the disease pathogenesis was two months, with daily use at the beginning of treatment (Fig. 1), which could be reduced to two or three times a week when the skin became thin and less inflamed. A satisfactory response was considered to be the thinning of the CHILD nevi, the reduction of pruritus and abrasions that predispose to infections, also facilitating adaptation for the use of prostheses in hypoplastic limbs, in addition to a reported improvement in quality of life.

**Fig. 1:**
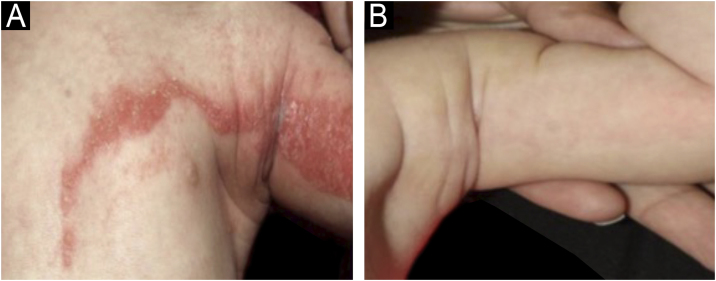
CHILD nevus, showing Blaschkoid lesion in fold area of ​​a newborn patient (a) without treatment (b) after two months of use of topical 2% Simvastatin + 2% Cholesterol.

Loss of efficacy was observed over the years, a fact not yet described, without improvement with the association of keratolytic agents or calcipotriol. Observing the principle of potency of pharmacological action of statins,^10^ lovastatin was replaced with other drugs with more potent action, such as 2% simvastatin and then 2% atorvastatin, when loss of efficacy was identified during follow-up and therapeutic response was re-established. Case 1 was treated with Lovastatin and then switched to Simvastatin, temporarily stabilized, but continues with irregular treatment and recrudescence of lesions. Case 2 required a third formulation, atorvastatin (Fig. 2). Case 3 has remained stable with lovastatin alone for eight years. Cases 4 and 5 were started with simvastatin, are stable, and also satisfied with the obtained results.

**Fig. 2:**
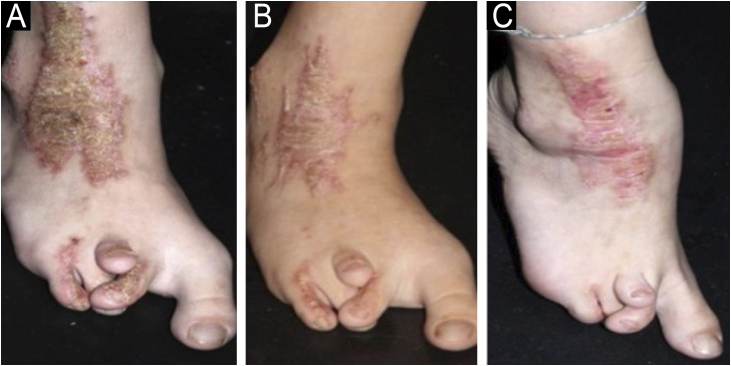
CHILD nevus on the right lower limb associated with dysplasia. (a) Recurring lesion after nine years of use of 2% Lovastatin + 2% cholesterol. (b) Partial improvement with replacement with 2% simvastatin + 2% cholesterol. (c) Significant improvement after replacement with topical 2% atorvastatin + 2% cholesterol.

Studies with larger sample sizes and long-term follow-up are needed to understand clinical evolution and establish effective therapeutic guidelines. This is the first publication with long-term follow-up of CHILD nevus treatment with statins, focusing on the long-term loss of response to the less potent lovastatin, and subsequent re-establishment of response with the use of topical simvastatin and atorvastatin, with satisfactory results for the patients.

## ORCID ID

Milene Tiburcio Narenti Ferradoza: 0000-0002-5864-7259

Ana Clara Maia Palhano: 0000-0002-0404-6482

Julia Maria de Oliveira Neumayer: 0009-0001-9651-1942

Luciana Paula Samorano: 0000-0001-7077-8553

Maria Cecilia Rivitti-Machado: 0000-0003-2910-7330

Zilda Najjar Prado de Oliveira: 0000-0002-8596-1999

## Financial support

None declared.

## Authors’ contributions

Milene Tiburcio Narenti Ferradoza: Design and planning of the study; collection, analysis, and interpretation of data; drafting and editing of the manuscript; approval of the final version of the manuscript.

Ana Clara Maia Palhano: Analysis and interpretation of data; drafting and editing of the manuscript; approval of the final version of the manuscript.

Julia Maria de Oliveira Neumayer: Analysis and interpretation of data; drafting and editing of the manuscript; approval of the final version of the manuscript.

Luciana Paula Samorano: Clinical intervention in the case; critical review of the manuscript; approval of the final version of the manuscript.

Maria Cecilia Rivitti-Machado: Effective participation in the research conception and orientation; critical review and approval of the final version of the manuscript.

Zilda Najjar Prado de Oliveira: Intellectual participation in the therapeutic conduct, critical review, and approval of the final version of the manuscript.

## Research data availability

Not applicable.

## Conflicts of interest

None declared.
